# Heart-rate independent myocardial T1-mapping using combined saturation and inversion preparation pulses

**DOI:** 10.1186/1532-429X-15-S1-P46

**Published:** 2013-01-30

**Authors:** Sebastian Weingärtner, Mehmet Akcakaya, Sophie Berg, Kraig V Kissinger, Warren J Manning, Reza Nezafat

**Affiliations:** 1 Department of Medicine, Beth Israel Deaconess Medical Center and Harvard Medical School, Boston, MA, USA; 2 Department of Radiology, Beth Israel Deaconess Medical Center and Harvard Medical School, Boston, MA, USA; 3 Computer Assisted Clinical Medicine, University Medical Center Mannheim, Heidelberg University, Mannheim, Germany

## Background

Myocardial T1 mapping remains a challenging task due to restrictions imposed by cardiac and respiratory motion. Modified Look-Locker Inversion Recovery (MOLLI) [1] is widely used for 2D cardiac T1-mapping. In MOLLI, the spin-lattice relaxation curve is sampled several times after a single magnetization preparation. The ECG triggered imaging induces a disturbance in the relaxation curve, which varies based on the heart rate. Hence, MOLLI T1 measurements show strong correlations to the heart rate especially in pre-contrast. We developed a novel T1 mapping sequence that enables heart-rate invariant myocardial T1 mapping.

## Methods

**Figure 1:**
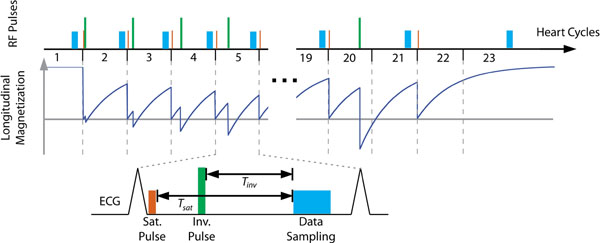
Sequence diagram depicting the SAPPHIRE T1-mapping sequence: a saturation pulse is performed after the R-wave to erase the magnetization history. It is followed by the inversion pulse and a single-shot image readout. To extend the range of applicable inversion times the data readout of some SAPPHIRE experiments is performed in the heart-cycle after the magnetization preparation. Additionally the first heart-cycle is performed without magnetization preparation and the last heart-cycle with the saturation pulse only. This increases the effective inversion times and improves the T1 fit.

Figure 1 shows the schematic of the proposed SAturation Pulse Prepared Heart rate independent Inversion-REcovery sequence (SAPPHIRE). A saturation pulse is inserted right after the R-wave of selected heart-cycles. This dephases the magnetization in the imaging volume and eliminates the need for recovery periods after the magnetization preparation. The saturation pulse is followed by an inversion pulse after a variable delay to create various T1 weighted contrasts in the images. Eleven SAPPHIRE images are acquired, where each magnetization preparation is followed by a single-shot imaging in the same heart-cycle. Six additional SAPPHIRE images are acquired with longer inversion times, by performing the data sampling in the heart-cycle after the magnetization preparation. The first heart cycle is performed without any prepulses, to provide a spin-density weighted image, which facilitates the T1-fit.

SAPPHIRE T1-mapping was compared to MOLLI in phantom measurements and in healthy volunteers. A bottle phantom with a T1 of ~1300 ms was imaged using both T1-mapping sequences at various simulated ECGs with different heart-rates. Furthermore, pre-contrast T1-maps in five healthy volunteers were acquired using SAPPHIRE T1-mapping and MOLLI.

## Results

**Figure 2:**
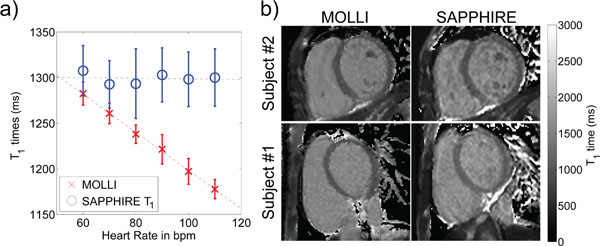
a) Calculated T1 values from phantom images acquired with different simulated R-R interval of 60-120 beats per minute. T1 measurements calculated from MOLLI shows linearly dependent on the duration of R-R, however SAPPHIRE T1 measurements were relatively constant. b) T1 maps from two healthy subjects acquired without gadolinium contrast showing higher homogeneity of the T1 measurements in SAPPHIRE.

In the phantom measurements SAPPHIRE T1-mapping is in good agreement with MOLLI measurements at a simulated heart-rate of 60 bpm (Relative difference: <2%). The SAPPHIRE T1-times, as depicted in Figure 2a), showed no significant correlation with the heart rate (r =-0.10), while MOLLI is highly correlated (r=-0.99). The T1 times in myocardium and the blood pool of the LV of the volunteers showed no significant difference between the two sequences (p = 0.20, p = 0.10). Figure 2b) shows exemplary T1-maps of two subjects. SAPPHIRE T1-mapping required slightly longer breath holds (16-23s SAPPHIRE vs. 12-17s MOLLI).

## Conclusions

SAPPHIRE T1-mapping enables heart rate independent myocardial T1-mapping. The heart-rate invariance is achieved by applying a combination of saturation and inversion pulses as magnetization preparation.

## Funding

Deutsche Telekom Stiftung; NIH:R01EB008743-01A2; NIH: K99HL111410-01

## References

[B1] Messroghli MRM 2008

